# *Drosophila* as a model in organophosphate toxicology

**DOI:** 10.1080/19336934.2026.2695497

**Published:** 2026-06-27

**Authors:** Marta Tkachuk, Nataliya Matiytsiv

**Affiliations:** Department of Genetics and Biotechnology, Ivan Franko National University of Lviv, Lviv, Ukraine

**Keywords:** Organophosphates toxicity, neurotoxicity, *Drosophila* model, NTE/SWS gene, OPIDN, SMART assay, genotoxicity

## Abstract

Organophosphates (OPs) cause acute cholinergic toxicity, oxidative stress, DNA damage, and delayed axonopathy through neuropathy target esterase (NTE) inhibition. Efficient *in vivo* models capturing this toxicity spectrum are essential for risk assessment and therapeutic discovery. Here, we review *Drosophila melanogaster* as a versatile platform for OP toxicology across: (1) wild-type assays using dietary and vapour exposures with behavioural endpoints (survival, locomotion, negative geotaxis) and biochemical markers (acetylcholinesterase activity, oxidative stress indices); (2) genotoxicity assessment via the Somatic Mutation and Recombination Test (SMART) and comet assay; and (3) the *swiss cheese* (*sws*) mutant model – the *Drosophila* ortholog of human PNPLA6/NTE – enabling investigation of organophosphate-induced delayed neuropathy (OPIDN) independent of cholinergic effects. Together, these approaches provide hazard characterization and support discovery of protective strategies targeting both cholinergic and non-cholinergic pathways.

## Introduction

Organophosphates (OPs) are a large class of phosphorus-containing esters used as insecticides, industrial additives, and nerve agents. Their canonical mechanism of acute toxicity is irreversible inhibition (or slowly reversible, depending on the compound and ‘aging’ state) of acetylcholinesterase (AChE), leading to accumulation of acetylcholine at synapses and cholinergic crisis [1]. A subset of OPs also can cause a delayed distal axonopathy known as organophosphate-induced delayed neuropathy (OPIDN), mechanistically linked to inhibition and ‘aging’ of neuropathy target esterase (NTE/PNPLA6) [2]. Beyond the acute cholinergic effects, organophosphates exert diverse toxic impacts – including oxidative stress, DNA damage, mitochondrial dysfunction, and disruption of lipid and signalling pathways – which vary by dose, compound, and exposure duration [3] (Table 1).

**Table 1.** Organophosphates studied in *Drosophila melanogaster* and their main reported effects.

Organophosphate	Exposure / life stage	Main reported effects in Drosophila	Key references
Chlorpyrifos	Dietary; larvae and adults; acute and chronic	AChE inhibition; oxidative stress (increased ROS, lipid peroxidation, altered SOD/CAT/GST); apoptosis and DNA damage; activation of p38 MAPK and Nrf2 signalling; hsp70 induction; reduced survival, fecundity and hatchability; coordination deficits and developmental delay after early-life exposure	[8,11–16]
Dichlorvos (DDVP)	Dietary (larvae); vapour/fumigation in adults (highly volatile)	AChE inhibition; dose- and time-dependent ROS generation; hsp70 induction; altered SOD, CAT and lipid-peroxidation markers; apoptosis; cellular damage in reproductive tissues; genotoxicity in the SMART wing-spot assay	[12,17]
Diazinon	Dietary; larvae	Genotoxicity in the SMART wing-spot assay — concentration-dependent induction of small and large single spots, consistent with somatic mutation and/or mitotic recombination	[17]
Methyl parathion	Dietary; larvae	Genotoxicity in the SMART wing-spot assay — concentration-dependent induction of mutant clones	[17]
Azamethiphos	Dietary; larvae	Genotoxicity in the SMART wing-spot assay — concentration-dependent induction of mutant clones	[17]
Acephate	Chronic sub-lethal dietary exposure from 1st-instar larvae through adulthood	ROS-mediated injury at organismal and sub-organismal levels (increased lipid peroxidation, altered catalase); apoptosis; altered male body weight; disrupted testis architecture, reduced germ-cell viability and number of mating pairs; altered expression of vitellogenin and mitoferrin	[18,19]
Tri-*ortho*-cresyl phosphate (TOCP / ToCP); major NTE aging component of technical TCP	Dietary; adult exposure followed by post-exposure observation	Only weak AChE inhibition; delayed behavioural deficits and neurodegeneration with vacuolisation appearing ~2 weeks after exposure; axonal degeneration in primary neurons within ~1 h *in vitro*; reduced PKA activity through aging-modified SWS/NTE; phenotypes overlap with *sws* loss-of-function — used to model OPIDN	[20]

AChE, acetylcholinesterase; ROS, reactive oxygen species; SOD, superoxide dismutase; CAT, catalase; GST, glutathione-S-transferase; SMART, somatic mutation and recombination test; OPIDN, organophosphate-induced delayed neuropathy; SWS, Swiss cheese; NTE, neuropathy target esterase.

Despite progressive regulatory restrictions on agricultural OPs, ongoing human exposure at low-to-moderate levels continues in occupational and community settings, and agents such as tricresyl phosphate (TCP) persist in aviation oils and other industrial contexts [4–6]. Constantly emerging chemicals should be tested quickly. Hence, *in vivo* model systems that resolve both acute cholinergic and chronic non-cholinergic effects remain essential for toxicology, risk assessment, and treatment discovery.

*Drosophila melanogaster* offers a cost-effective, whole-animal platform to investigate OP toxicity over the lifespan. Advantages include short generation time, well-annotated genetics, conserved neurotransmission and stress-response pathways, and a deep toolkit for behavioural, physiological, and genotoxic readouts that work in wild-type backgrounds and in specialized lines [7]. *Drosophila* has enabled significant progress in the search for new treatments and protective strategies against organophosphate (OP) toxicity. Key achievements include the identification of plant-derived compounds, probiotics, and novel acetylcholinesterase (AChE) reactivators as potential therapeutic agents [8–10]. This paper outlines the fundamental considerations for approaching toxicity studies of organophosphates with *Drosophila* for researchers entering the field. Below, we summarize methods and findings from studies using wild-type flies for exposure, specialized genetic lines to dissect genotoxic mechanisms, and the SWS/NTE neuropathy model to test TCP and other OPIDN-inducing organophosphates.

### Use of wild-type flies in organophosphate exposure studies

Like humans, *Drosophila* exhibit broad variation in outcomes associated with toxicant exposures, and it is therefore critical to consider how genetic background can influence tolerance and susceptibility to OPs. While the ease of controlling exposures and executing medium-throughput assays makes the fly optimal for comparative studies across toxic agents, interpretations of behavioural and biomarker assays should also consider investigating more than one wild-type strain to confirm the robustness and consistency of the toxicity mechanism under investigation. Commonly used wild-type backgrounds in *Drosophila* toxicology include *Oregon R*, *Canton-S*, and *Berlin K*; in addition, the *Drosophila* Genetic Reference Panel (DGRP) – a set of ~ 200 sequenced inbred lines derived from a natural population – provides a particularly powerful resource for systematically dissecting how natural genetic variation modulates OP responses [21]. The full panel is publicly available through the Bloomington *Drosophila* Stock Centre [22], https://bdsc.indiana.edu/stocks/wt/dgrp.html].

## Methods of exposure

Since there are volatile and non-volatile OPs, there are two ways to administer OP to *Drosophila*. Dietary administration mixes a defined OP concentration into standard food media before solidification or delivers OP in liquid food using capillaries (the CAFE assay, applied to adults); this allows dose – response and intake quantification across larvae or adults [23–25].

Second, adult exposures can be achieved via contact or vapour (fumigation) where OPs with high volatility (like dichlorvos) are delivered in enclosed arenas; while less common for agricultural OPs, vapour paradigms are established in entomology for toxicant delivery and are adaptable to *Drosophila* [26]. However, for most insecticidal OPs (chlorpyrifos, diazinon, acephate) and tricresylphosphate, food-based regimens predominate. Developmental windows (embryo – larva – pupa) and chronic low-dose adult exposures are routinely implemented to model subclinical neurotoxicity.

Both acute (hours to days) and chronic (days to weeks) exposures are used, with developmental and adult stages offering distinct insights. Acute assays are used for rapid screening of toxicity, dose-response, and lethality, often in larvae or adults. These are suitable for identifying highly toxic compounds and for studies of immediate effects. Chronic exposures are essential for modelling subclinical neurotoxicity, developmental effects, and long-term outcomes, especially mimicking environmental or occupational exposures. This is commonly used for less volatile, food-administered OPs like chlorpyrifos and diazinon.

## Behavioural and physiological tests

Behavioural assays in wild-type flies aim to capture OP-induced neurotoxicity. General toxicity can be monitored in forms of total survival (from embryo to adult) as well as partial survival scores (embryo to pupae, pupae to adult survival), providing population-level sensitivity. Changes in pupation height can also be measured, indicating developmental toxicity and whether exposure impairs larval locomotion, motivation, or nervous system function [16]. Negative geotaxis (startle-induced climbing/RING) quantifies locomotor vigour over time; high-resolution gait analysis (e.g. FlyWalker) detects subtle coordination deficits after developmental chlorpyrifos exposure [27,28]. Additional endpoints include sleep/activity monitoring, courtship, reproduction metrics (fecundity, hatchability), and learning paradigms where cholinergic and oxidative stress loads impact performance.

Physiological markers track mechanism of action and systemic stress. An important physiological distinction must be noted here: unlike mammals, which use acetylcholine (ACh) at the neuromuscular junction, *Drosophila* uses glutamate for fast excitatory neuromuscular transmission [29], while ACh is the primary excitatory neurotransmitter in the central nervous system [30]. Consequently, adult heads – which contain the cholinergic brain – are the appropriate tissue for assaying AChE activity in fly OP studies. AChE activity in head homogenates is routinely inhibited by OPs and restored by efficacious oxime reactivators, validating *Drosophila* for discovery/triage of medical countermeasures [10]. Reproductive endpoints (fecundity, hatchability) and developmental timing are sensitive to sublethal chlorpyrifos and acephate, including transgenerational effects under chronic exposure [11,12,19].

## Biochemical markers

Key wild-type biomarkers include: biochemical inhibition of AChE with or without reactivation, oxidative stress and mitochondrial metrics, apoptosis and DNA damage (e.g. TUNEL, γH2AX, comet). For example, oxidative stress markers (ROS, lipid peroxidation, thiols), mitochondrial viability, and antioxidant enzymes (SOD, catalase, GST) shift under chlorpyrifos or acephate, along with DNA damage and apoptosis in larval tissues at higher doses [13,15,18].

**Figure 1.**
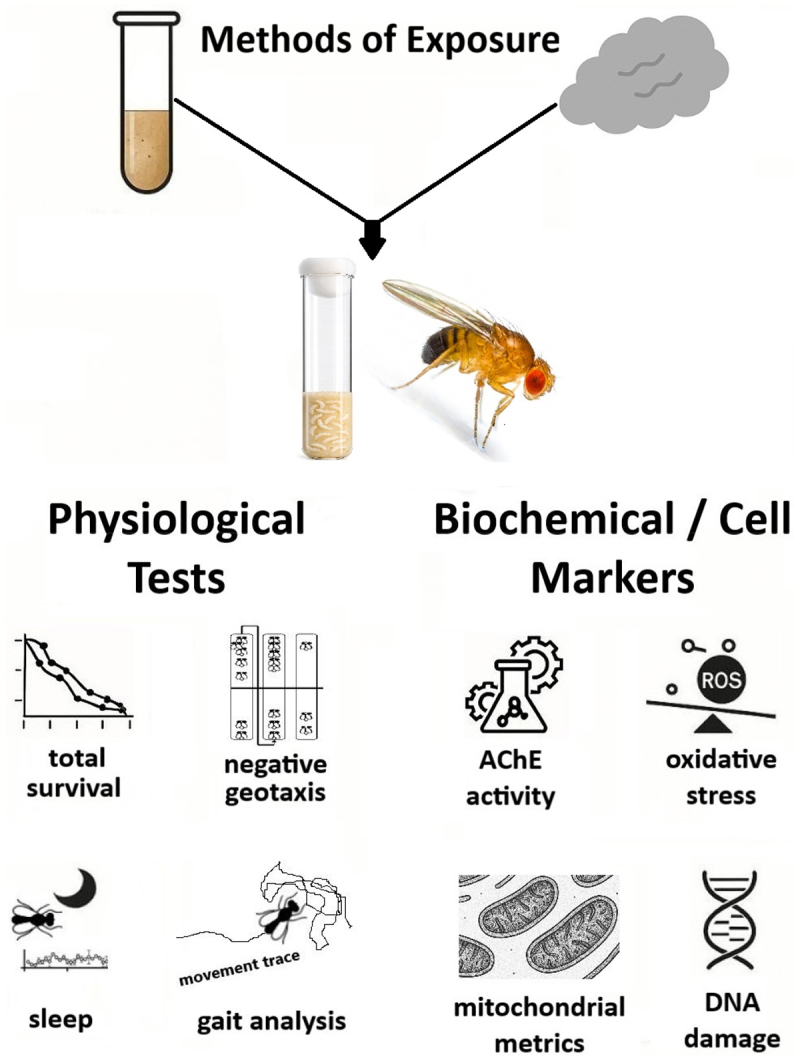
A summary illustration depicts a typical experimental design in *Drosophila* organophosphate studies — from exposure method (dietary or vapor) to behavioral, physiological and cell markers.

The use of *Drosophila* allows us to study the effects of OPs at all stages, on various physiological and cellular systems (Figure 1). These markers are feasible without transgenic tools, scale to medium-throughput screening, and translate to vertebrate outcomes.

### Use of specialized genetic lines in genotoxicological studies of organophosphate exposure

**Figure 2.**
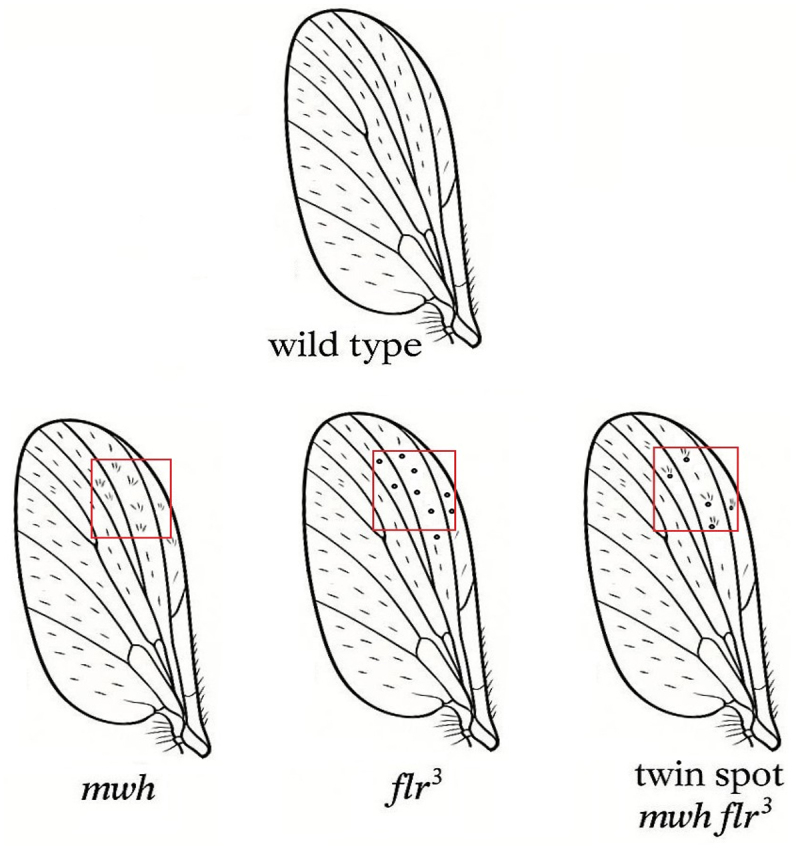
Schematic representation of the *Drosophila* wing SMART test. Mutations or recombination events in developing wing cells produce clones of recessive marker cells (*mwh, flr^3^*), appearing as spots on the adult wing surface. This method distinguishes recombination-derived from mutation-induced genotoxic events.

Because OPs can affect cholinergic synapses, axon maintenance pathways, and genome integrity, *Drosophila* genetics is invaluable for mechanistic dissection. The Somatic Mutation and Recombination Test (SMART, or a wing-spot test) uses recessive markers such as multiple wing hairs (*mwh*) and flare (*flr^3^*) to visualize mitotic recombination, mutation, deletion, or chromosome loss as clone spots in adult wings (Figure 2). Larvae, heterozygous for specific recessive markers (e.g. *mwh, flr^3^*) are exposed to test substances. Genetic damage (mutation or recombination) in the developing wing disc cells leads to clones of mutant cells, which appear as spots on the adult wing surface. SMART detects loss of heterozygosity and distinguishes recombination-driven from mutation-driven events with crossing schemes and balancers, and has been validated across laboratories and chemicals. The presence of twin spots (adjacent mwh and flr^3^ clones) is a hallmark of mitotic recombination, as these arise only when a recombination event occurs between homologous chromosomes. Single spots can result from either mutation or recombination. However, by using different genetic crosses (marker-heterozygous vs. balancer-heterozygous), researchers can distinguish the origin. In balancer-heterozygous flies, only mutations can produce spots, as recombination is suppressed [31,32]. OPs including malathion, diazinon and others induce small and large single spots in SMART, consistent with genotoxic or recombinogenic potential at sufficiently high exposures [17].

Complementary assays strengthen genotoxic inference. The alkaline comet assay has been adapted to larval tissues (brain, midgut, haemolymph, imaginal discs) *in vivo* and in S2 cells *in vitro*, enabling direct measurement of strand breaks and repair kinetics. In *Drosophila*, comet readouts can be combined with repair-deficient mutants (e.g. mus209/PCNA) to map pathway usage and OP-specific lesion processing [33,34]. In addition, γH2AX immunostaining (a marker of DNA double-strand breaks) and apoptosis markers (caspase activation, DNA laddering) have been applied after chlorpyrifos exposure, linking oxidative injury to DNA damage [14].

### The SWS/NTE neuropathy model and the effects of tricresyl phosphate (TCP)

A subset of organophosphates (OPs), most notably tri-ortho-cresyl phosphate (ToCP), a key component of technical tricresyl phosphate (TCP) mixtures, can induce organophosphate-induced delayed neuropathy (OPIDN) in susceptible species. OPIDN is mechanistically linked to the inhibition and subsequent ‘aging’ of neuropathy target esterase (NTE), a membrane-associated phospholipase critical for phosphatidylcholine metabolism and axonal maintenance. In Drosophila, the *swiss cheese* (*sws*) gene encodes the NTE ortholog, sharing high sequence and functional conservation with vertebrate NTE [20,35,36].

Loss-of-function *sws* mutants in *Drosophila* exhibit progressive behavioural deficits and neurodegeneration in both neurons and glia, closely mirroring the axonopathy seen in human NTE-related disorders. SWS localizes to the endoplasmic reticulum and lipid droplets, playing a central role in phospholipid homoeostasis. Notably, neuron-specific or glial expression of human PNPLA6/NTE in *sws* mutants can rescue neurodegenerative phenotypes, underscoring the evolutionary conservation of function [36].

Previous studies have shown that mutations in *sws* cause a wide range of neurodegenerative phenotypes, including locomotor impairments and glial pathology reminiscent of human NTE-related neuropathies. These *sws^1^* lines therefore provide a valuable complementary tool for comparison with organophosphate exposure models. Characterization of *sws* phenotypes can inform potential targets of OP toxicity, particularly in relation to lipid signalling, oxidative stress, and cell death pathways. Studies revealed that similar lipid and morphological changes may underlie OP-induced neurodegeneration [37,38].

*Drosophila* model have been instrumental in dissecting the effects of TCP/ToCP exposure. Experiments combine developmental or adult TCP exposure with behavioural assays, histopathology (vacuolization, axon integrity) and lipidomic profiling (elevated lysophosphatidylcholine and lysophosphatidic acid) to test the disruption of lipid signalling following NTE inhibition and ageing. Importantly, TCP/ToCP causes only weak acetylcholinesterase inhibition, allowing *Drosophila* sws model to distinguish delayed axonopathy from acute cholinergic toxicity. Integration of this methods with genotoxicity assays (SMART, comet) enables parallel assessment of neurotoxicity and genotoxicity under TCP exposure, providing comprehensive hazard profile.

The *sws^1^* mutants serve as an important additional control, as they lack the primary OP target, NTE. This absence allows for discrimination between general toxic effects and those specifically mediated by NTE inhibition. We therefore propose integrating experiments on both wild-type and sws flies to achieve a more comprehensive interpretation of OP toxicity mechanisms and to facilitate testing of potential protective strategies.

Phenotypic manifestations observed in *sws* mutants may guide the direction of organophosphate toxicity research by indicating which cellular pathways – such as phospholipid metabolism, axonal maintenance, or glial support – are most sensitive to NTE dysfunction and thus represent potential targets of OP action. Comparison of these mutant phenotypes with those induced by organophosphate exposure can help to distinguish NTE-dependent from NTE-independent effects, highlighting whether observed neurodegenerative outcomes arise from specific inhibition of the NTE pathway or from broader toxic mechanisms.

## Conclusions and outlook

*Drosophila melanogaster* is a powerful *in vivo* platform for OP toxicology. Wild-type assays detect neurotoxicity with scalable behavioural and biochemical readouts; genetic lines and SMART/comet assays resolve genotoxic mechanisms; and the SWS/PNPLA6 models OPIDN-relevant axonopathy and TCP effects. Together, these approaches enable mechanism-based risk assessment, comparative toxicology across OPs, and discovery of protective strategies beyond AChE reactivation – particularly interventions that stabilize axonal lipid homoeostasis and stress responses. Future work should expand multi-omics (lipidomics, metabolomics) under chronic low-dose exposure, and systematically bridge *Drosophila* endpoints to vertebrate and human data.
